# Using Quadratic Programming to Reconstruct Data From Published Survival and Competing Risks Analyses

**DOI:** 10.1002/sim.70474

**Published:** 2026-03-03

**Authors:** Andrew C. Titman

**Affiliations:** ^1^ School of Mathematical Sciences Lancaster University Lancaster Lancashire UK

**Keywords:** competing risks analysis, pseudo‐individual patient data, quadratic programming, survival analysis

## Abstract

The ability to retrieve pseudo‐individual patient data (IPD) from published survival study results is important to facilitate meta‐analysis, evidence synthesis or secondary data analyses for the purpose of decision modeling for cost effectiveness analysis. While established methods exist for retrieving pseudo‐IPD from Kaplan–Meier plots, these algorithms are not easily extendable to other types of survival data, nor do they allow all available information to be incorporated. An optimization‐based approach is proposed where the task of reconstructing the IPD is formulated as a quadratic program (QP) with linear constraints. The method easily allows auxiliary information such as marked censoring times. Moreover, the same approach can be used to reconstruct patient‐level competing risks survival data from published cumulative incidence functions. In simulation, the QP‐based method is shown to outperform existing algorithms particularly when data on numbers at risk and marked censoring times are available. The methods are illustrated through reconstruction of data from a published study on patients with advanced stage follicular lymphoma.

## 1 Introduction

It is often necessary or desirable to obtain approximate individual‐level patient data (IPD) from a published study for the purposes of meta‐analysis or evidence synthesis. For instance, meta‐analysis procedures that use IPD may have superior properties to those relying only on summary level data [1]. Similarly, a published paper may not provide the required estimate or summary statistic for a particular analysis but clearly this could be computed were the IPD available. The availability of IPD also facilitates parametric modeling of the data, which can be important for survival extrapolation in cost‐effectiveness modeling. Nevertheless, it is often not possible to obtain the original data for reasons of patient or commercial confidentiality, or the process of obtaining the original data is too time consuming. In the context of time‐to‐event outcomes, the Kaplan–Meier estimate of the survivor function, which is almost always reported, conveys a substantial amount of information particularly if information on the number of patients at risk at some time points is also given. This has led to several proposals for algorithms to extract summary statistics or reconstruct *pseudo individual patient data* (pseudo‐IPD) from the Kaplan–Meier curve [2, 3, 4, 5].

By far the most commonly used method for generating pseudo‐IPD is that of Guyot et al., referred to as the iKM algorithm [6]. This involves eight distinct steps, where initial estimates of the number at risks, number of observed events and number censored at each time point are made and then subsequently refined. More recently Liu et al. [7] proposed a modified iKM algorithm, which improves the robustness and stability of the original iKM algorithm and is implemented within the *IPDfromKM* package in R [8]. Separately, Rogula et al. [9] have proposed an algorithm of a similar nature to iKM which uses the information from the marked censoring times, or “tick” marks, often displayed on published Kaplan–Meier curves. In simulation they show that this approach usually performs better than iKM when these tick marks are available. However, their method does not attempt to use information of total number of events or numbers at risk at intermediate times to refine the estimate.

While these existing algorithms have been shown to perform well in a wide range of situations, the underlying algorithms are somewhat ad hoc in nature, which makes their modification to accommodate additional information, or their extension to other types of event history data, difficult.

Typically, the time points and survival decrements obtained through digitization will be subject to varying levels of error or omission depending on the image quality and the digitization method used. In contrast, it is usually reasonable to assume that the reported numbers of patients at risk and total number of events will be accurate. However, while the iKM and modified iKM algorithms use the information, they do not necessarily guarantee that the pseudo‐IPD obtained will agree in terms of number of patients at risk and total number of events. Conversely, the method of Rogula et al. does not use this information at all.

Many time‐to‐event endpoints are subject to competing risks, meaning that the appropriate quantity of interest should be the cumulative incidence function (CIF) with respect to the event(s) of interest [10]. While non‐parametric estimates of the CIFs of a similar nature to the Kaplan–Meier estimates can be produced, and are routinely reported in studies involving competing risks, there is currently no established method of reconstructing IPD from such estimates.

This paper presents a general framework for reconstructing IPD by expressing the procedure as a quadratic programming optimization problem. The remainder of the paper is as follows. Section 2 presents the quadratic programming framework in the context of reconstructing IPD from Kaplan–Meier curves. In Section 3, the approach is extended to provide a method to extract IPD from published cumulative incidence distribution curves in competing risks analyses. The methods are illustrated on published results from a study into long‐term outcomes of patients with advanced follicular lymphoma in Section 4, and assessed via simulation in Section 5. The paper concludes with a discussion including consideration of potential extensions of the approach.

## 2 Quadratic Programming Framework

The overall aim of reconstructing the IPD is to find a set of data points that will return an estimate of the survival or cumulative incidence curve as close as possible to the observed estimate. As such, it can be viewed as an optimization procedure. In what follows, the problem is posed in terms of a quadratic program. Quadratic programming refers to optimization problems involving quadratic objective functions, for which there are efficient and well‐established algorithms for solving quadratic programs with linear constraints [11]. Such problems can be defined as finding the vector x of length p that will

(1) $$\begin{array}{l} \;\mathrm{minimize}\;\frac{1}{2}x^{\prime }\mathrm{Qx}+q^{\prime }x\\ \text{subject to}\;\mathrm{Ax}\leq b \end{array}$$

where Q is a p×p symmetric matrix, q is a vector of length p, A is an m×p matrix and b is a vector of length m.

We look to frame the problem of finding the number of events and number of patients censored at individual time points as a linearly constrained optimization problem, where the objective is to minimize the squared discrepancy between the observed decrement of the survivor function and the decrement implied by completed data. For competing risks data, the objective becomes the minimization of the squared discrepancy between the increments of the cumulative incidence curve and the increments implied by the completed data. Information provided on the number of patients at risk and total number of events is used to define linear constraints for the quadratic program.

### 2.1 Kaplan–Meier Survival Data

First consider the case discussed by previous authors of reconstructing IPD from a published Kaplan–Meier curve and the number of patients at risk at certain time points. In this case, the available information consists of extracted survival curve estimates $\left\{s_{i},i=1,\ldots ,N_{s}\right\}$ at corresponding times $\left\{t_{i},i=1,\ldots ,N_{s}\right\}$ (representing co‐ordinates from the digitized Kaplan–Meier curve) and numbers at risk $\left\{R_{j},j=0,1,\ldots ,N_{r}\right\}$ at corresponding times $\left\{\tau_{j},j=0,1,\ldots ,N_{r}\right\}$ where $\tau_{0}=0$ and $R_{0}=N$ is the total number of patients at risk at time 0 (taken from information typically given below the time axis of the plot) where usually $N_{r}\ll N_{s}$. We assume that at any given time $t_{i}$, an unknown number of events, $d_{i}$, occurred and an unknown number of patients, $c_{i}$, were censored. Since a good reconstruction would ensure a close match between the “observed” curve defined by $s_{i}$ and the “fitted” curve defined through the $d_{i}$ and $c_{i}$, the aim is to minimize some measure of this discrepancy. The definition of the Kaplan–Meier estimator ensures that for all i we have the relationship

$$\frac{s_{i}}{s_{i-1}}=1-\frac{d_{i}}{r_{i}}$$

where $r_{i}=N-\sum_{j<i}\left(d_{j}+c_{j}\right)$ is the number at risk and $s_{0}=1$. Let $o_{i}=1-s_{i}/s_{i-1}$ then under a perfect reconstruction

(2) $$o_{i}\left(N-\sum_{j<i}\left(d_{j}+c_{j}\right)\right)-d_{i}=0$$

for all $i=1,\ldots ,N_{s}$. This motivates using a criterion

(3) $$W=\sum_{i=1}^{N_{s}}{\left(o_{i}\left\{N-\sum_{j<i}\left({\tilde{d}}_{j}+{\tilde{c}}_{j}\right)\right\}-{\tilde{d}}_{i}\right)}^{2}$$

to judge the fit of a given reconstruction with estimated event counts ${\tilde{d}}_{j}$ and ${\tilde{c}}_{j}$. This particular formulation is useful since each term to be squared in (3) is linear in the variables to be optimized (${\tilde{d}}_{i}$ and ${\tilde{c}}_{i}$) and hence W is a quadratic form. Given the information on patients at risk, we have equality constraints of the form.

(4) $$\sum_{i\in C_{j}}\left({\tilde{d}}_{i}+{\tilde{c}}_{i}\right)=R_{j}-R_{j-1},\text{for}\;j=1,\ldots ,N_{r}$$

where $C_{j}=\left\{i:\tau_{j-1}\leq t_{i}<\tau_{j}\right\}$. Note that, in some cases there may be no uncensored events between $\tau_{j-1}$ and $\tau_{j}$, but nevertheless $R_{j}-R_{j-1}>0$. To ensure that the equality constraints can be met in these cases, we include $\left\{\tau_{1},\ldots ,\tau_{N_{r}}\right\}$ in the set of candidate times at which a patient could be censored. This assumption differs from that of the iKM and modified iKM algorithms where only the decrement points are used to determine the censoring times. If information on the total number of events, $N_{E}$, is available a further equality constraint

$$\sum_{i=1}^{N_{s}}{\tilde{d}}_{i}=N_{E},$$

should be included. In addition, since the Kaplan–Meier curve can only decrease at times corresponding to at least one observed event, we have inequality constraints ${\tilde{d}}_{i}\geq 1$ for $i\in \left\{j:o_{j}>0\right\}$ and ${\tilde{c}}_{i}\geq 0$ for $i=1,\ldots ,N_{s}$.

Hence, ignoring the further condition that the ${\tilde{d}}_{i}$ and ${\tilde{c}}_{i}$ are integers, the minimization problem can be expressed as a quadratic program with linear constraints for which it is straightforward to find the solution with standard software. For instance, in ***R*** the function *solve.QP* within the package *quadprog* will perform such optimization [12]. The explicit form of the quadratic program in terms of the quantities given in (1) is given in Appendix A.

Any proper reconstruction of the data requires that ${\tilde{d}}_{i}$ and ${\tilde{c}}_{i}$ are integers. If these integer constraints are imposed, then the formal minimization problem becomes substantially more difficult. The problem is then a mixed integer quadratic program (MIQP) which can be solved, for instance, via either branch and bound or outer‐approximation algorithms [13, 14]. Currently most software that can accommodate MIQP is commercial, although IBM‐CPLEX [15] is available free for academic use and can be used within ***R*** using the *Rcplex* [16] or *cplexAPI* [17] packages. The main practical issue is that a MIQP is an NP‐hard problem [18] for which no polynomial time algorithm is available and hence the computation time required to find the optimum can become prohibitive, particularly for curves with a large number of identified points.

For practical purposes, one can specify the maximum allowable computation time for the MIQP solver, with software giving the best solution found in that time, rather than the global optimum. Alternatively, it is straightforward to devise a heuristic that maps the continuous solution to an approximate integer solution. In principle, one can either first round the number of observed events, $d_{i}$, and then determine the censored observations $c_{i}$ or instead round the censored observations first. Since for most time points $d_{i}\ll r_{i}$, the overall Kaplan–Meier estimate is more heavily influenced by changes in $d_{i}$ than $c_{i}$. Hence, an approach that rounds the $d_{i}$ first is considered. Let ${\tilde{d}}_{i}$ be the continuous solution for the number of events $d_{i}$. Then an integer estimate can be obtained through midpoint rounding of the cumulative sum of the observed events,

$${\hat{d}}_{i}=\left\lfloor \frac{1}{2}+\sum_{j=1}^{i}{\tilde{d}}_{j}\right\rfloor -\left\lfloor \frac{1}{2}+\sum_{j=1}^{i-1}{\tilde{d}}_{j}\right\rfloor .$$

Since the continuous solution still constrains the aggregate number of events to be integer, the solutions often involve a total of m+k events over m potential time points. The effect of the rounding scheme is to distribute the k events across these m times. However, the rounding potentially causes the ${\hat{d}}_{i}$ to violate the period constraints in (4). Let ${\tilde{c}}_{i}$ be the continuous solution for the number of censoring events then the equality constraints can be retained if, for each $i\in C_{j}$, the number of censoring events are scaled by

$$V_{j}=\frac{\left(R_{j}-R_{j-1}\right)}{\sum_{i\in C_{j}}\left({\hat{d}}_{i}+{\tilde{c}}_{i}\right)},j=1,\ldots ,N_{r}.$$

Hence, an integer solution can be obtained by taking

(5) $${\hat{c}}_{i}=\left\lfloor \frac{1}{2}+\sum_{k=1}^{i}\;V_{j\left(k\right)}{\tilde{c}}_{k}\right\rfloor -\left\lfloor \frac{1}{2}+\sum_{k=1}^{i-1}\;V_{j\left(k\right)}{\tilde{c}}_{k}\right\rfloor ,$$

where $j\left(k\right)=\left\{j:k\in C_{j}\right\}$ denotes the period within which time point k lies.

#### 2.1.1 Incorporating Information on Censoring Times

A particular advantage of the quadratic programming approach is the ease with which other information can be incorporated into the reconstruction. Published Kaplan–Meier curves often include marked censoring times, or “tick” marks, to indicate the times at which at least one patient was censored. If it can be assumed that the digitization captures all of these points then it is desirable to constrain the data reconstruction to only permit censoring events at those time points. Conversely, unless the tick point coincides with a decrement in the Kaplan–Meier curve, it is also sensible to constrain the number of survival events at those times to be 0. This can be achieved by augmenting the existing set of time points, $t_{i}$, to include the times of the tick marks. The value of ${\tilde{d}}_{i}$ at any time point added will be constrained to 0. Let $T^{*}$ be the set of tick marks, then for $C^{*}=\left\{i:t_{i}\in T^{*},o_{i}=0\right\}$ and $S^{*}=\left\{i:t_{i}\notin T^{*}\right)\}$ we add the additional constraints

$${\tilde{c}}_{j}=0\;\;\text{for}\;\;j\in S^{*},\;\text{and}\;\;{\tilde{d}}_{j}=0,\;\text{for}\;\;j\in C^{*},$$

which is equivalent to removing those variables from the optimization. Note also that, since in the continuous solution any j not corresponding to a tick mark would have ${\tilde{c}}_{j}=0$, this property would be retained after the scaling taken in (5). Depending on the confidence in the identification of the tick marks, one can make the further constraint that ${\tilde{c}}_{j}\geq 1\;\text{for}\;j\in C^{*},$ that is that all tick marks correspond to a distinct censoring time.

### 2.2 Avoiding Indeterminable Solutions

The resulting quadratic program may not necessarily have a positive definite objective matrix, Q, implying there is not a unique solution, even to the continuous problem. A notable, though not unique, reason for this issue is if tick marks are available and there are two or more tick marks between event times. Since the Kaplan–Meier estimate only depends on the numbers at risk at the event times, any allocation of the same number of censored events in those tick marks will result in the same decrements of the Kaplan–Meier estimate and hence the same objective value for the quadratic program. One possible solution to this issue would be to collapse together such points within the optimization, including some constraint that the number of censored events in the group should be at least the number of tick marks, and then use some heuristic to allocate events within groups of points (e.g., allocating equally).

Alternatively, one can instead add an additional term $\epsilon \left(\sum_{i}c_{i}^{2}\right)$ to the objective in (3) for some suitably small value ϵ – which leads to a preference for solutions with fewer censoring ties in cases where there is no, or virtually no, difference between solutions. This is similar to the iKM algorithm's default assumption that censoring events are uniformly distributed across decrement points. Considerations of the size for ϵ are mainly to ensure it is large enough such that the resultant objective matrix is deemed to be numerically invertible. If the approximate optimization method, based on integer rounding the continuous solutions is used, the resulting solution is not sensitive to the choice of ϵ and a value of ϵ=0.001 is a reasonable default choice. This latter approach of regularizing the optimization appears to perform better at reconstructing the pattern of censoring for both real and simulated datasets, and has the advantage of always ensuring the objective is positive‐definite.

## 3 Competing Risks Data

Often a time‐to‐event outcome may be subject to competing risks, for instance cohort studies into time‐to‐diagnosis of cancer would have death before cancer diagnosis as a competing risk. Such data can be characterized by the time‐to‐event T and the corresponding event indicator D∈{1,…,m} representing which of m possible causes occurred. The primary quantity of interest is usually the cumulative incidence function for the cause of interest, defined as $F_{j}\left(t\right)=P\left(T\leq t,D=j\right)$. Note that, unlike in the standard survival case, in the presence of competing risks the cumulative incidence will not generally correspond to one minus the Kaplan–Meier curve (i.e., computed by treating other events as censoring), but is instead be a function of all the individual cause‐specific hazards (see for instance, Putter et al. (2007) [10]).

The Aalen‐Johansen estimate of $F_{j}\left(t\right)$ is given by

(6) $$F_{j}\left(t\right)=\sum_{i:t_{i}\leq t}\frac{d_{\mathrm{ij}}}{r_{i}}\hat{S}\left(t_{i}-\right)$$

where

(7) $$\hat{S}\left(t_{i}-\right)=\prod_{k:t_{k}<t}\left(1-\frac{d_{k}}{r_{k}}\right)$$

is the Kaplan–Meier estimate of all‐cause survival just before time $t_{i}$ and $d_{k}=\sum_{j}d_{\mathrm{kj}}$ is the total number of events at time $t_{k}$.

Suppose that for a given study the non‐parametric cumulative incidence curves for each of m competing risks are available at a series of time points, such that there are observations $f_{\mathrm{ij}}$ at time $t_{i}$ for $i=1,\ldots ,N_{S}$ and j=1,…,m. Note that the assumption that ${\hat{F}}_{j}\left(t\right)$ is constant between observed points allows $f_{\mathrm{ij}}$ to be defined for all j even if the individual digitization of ${\hat{F}}_{j}\left(t\right)$ did not include an observation at $t_{i}$.

From (6) and (7) we can note that

$$\frac{f_{\mathrm{ij}}-f_{i-1j}}{1-\sum_{j}f_{i-1j}}=\frac{d_{\mathrm{ij}}}{r_{i}}$$

where $f_{0j}=0$. Using a similar argument to Section 2.1, taking

(8) $$o_{\mathrm{ij}}=\frac{f_{\mathrm{ij}}-f_{i-1j}}{1-\sum_{j}f_{i-1j}}$$

provides a set of identities $o_{\mathrm{ij}}r_{i}=d_{\mathrm{ij}}$ for $i=1,\ldots ,N_{S}$ and j=1,…,m and thus a quadratic form for the objective function can be obtained by

(9) $$W=\sum_{i=1}^{N_{S}}\sum_{j=1}^{m}{\left(o_{\mathrm{ij}}\left\{N-\sum_{k<i}\;\sum_{l=1}^{m}d_{\mathrm{kl}}-\sum_{k<i}c_{k}\right\}-d_{\mathrm{ij}}\right)}^{2}.$$

As in the Kaplan–Meier case, we would look to add an additional term $\epsilon \left(\sum_{i=1}^{N_{S}}c_{i}^{2}\right)$ to (9) to ensure the objective matrix is positive definite. Note that (9) reduces to (3) when there is a single risk (and hence $F_{1}\left(t\right)\equiv 1-S\left(t\right)$).

The information on patients at risk defines equality constraints of the form

$$\sum_{i\in C_{j}}\;\sum_{k=1}^{m}\left\{d_{\mathrm{ik}}+c_{i}\right\}=R_{j}-R_{j-1},j=1,\ldots ,N_{r}$$

where $C_{j}=\left\{i:\tau_{j-1}\leq t_{i}<\tau_{j}\right\}$. If available, information on the total number of events of each type, $N_{\mathrm{Ej}}$, could be incorporated through m additional equality constraints

$$\sum_{i=1}^{N_{S}}d_{\mathrm{ij}}=N_{\mathrm{Ej}},\text{for}\;j=1,\ldots ,m.$$

Alternatively, if only the total number of events of all types is known, an equality constraint, $\sum_{i=1}^{N_{S}}\sum_{j}^{m}d_{\mathrm{ij}}=N_{E},$ can be given. Since ${\hat{F}}_{j}\left(t\right)$ can only increase at times corresponding to an observed event of type j, we can also impose the inequality constraints $d_{\mathrm{ij}}\geq 1$ for $i\in \left\{k:o_{\mathrm{kj}}>0\right\}$.

As in the case of Kaplan–Meier survival data, either an “exact” MIQP can be solved or else the method may proceed by first solving the continuous QP and then applying a heuristic to approximate the integer solution. Specifically, for continuous solution ${\tilde{d}}_{\mathrm{ij}}$ the integer solution is found by taking

$${\hat{d}}_{\mathrm{ij}}=\left\lfloor \frac{1}{2}+\sum_{k=1}^{i}{\tilde{d}}_{\mathrm{kj}}\right\rfloor -\left\lfloor \frac{1}{2}+\sum_{k=1}^{i-1}{\tilde{d}}_{\mathrm{kj}}\right\rfloor .$$

The estimated censoring events are then found in the same way as in Section 2.1, by using the midpoint rounding in (5) with scaling

$$V_{j}=\frac{\left(R_{j}-R_{j-1}\right)}{\sum_{i\in C_{j}}\left(\sum_{k=1}^{m}{\hat{d}}_{\mathrm{ik}}+{\tilde{c}}_{i}\right)},j=1,\ldots ,N_{r}.$$

Note that if only one risk is of interest, provided the all‐cause Kaplan–Meier curve and the cumulative incidence function for the risk of interest are reported, it is possible to recover the sufficient statistics to either perform a Cox proportional cause‐specific hazard [19] or Fine‐Gray competing risks analysis [20] with respect to the cause of interest.

## 4 Illustrative Example: Advanced Follicular Lymphoma Study

As an illustrative example, the results from a retrospective study on long‐term outcomes of patients with advanced stage follicular lymphoma (FL) are considered [21]. The authors followed a cohort of 286 stage III‐IVA FL patients seen between 2000 and 2011 who followed a “watch and wait” management strategy. A particular endpoint of interest was progression‐free survival (PFS) which was defined as the time from diagnosis to lymphoma treatment or death from any cause.

### 4.1 Progression‐Free Survival Data

In the original paper, the Kaplan–Meier estimate of progression free‐survival among the ‘watch and wait’ management group is presented, including information on the number of patients at risk at 20‐month intervals and tick marks to indicate the times at which patients were censored. Digitization of the curves was performed by using WebPlotDigitizer [22], identifying 113 distinct decrement points and 80 tick marks via the manual extraction method.

**FIGURE 1:**
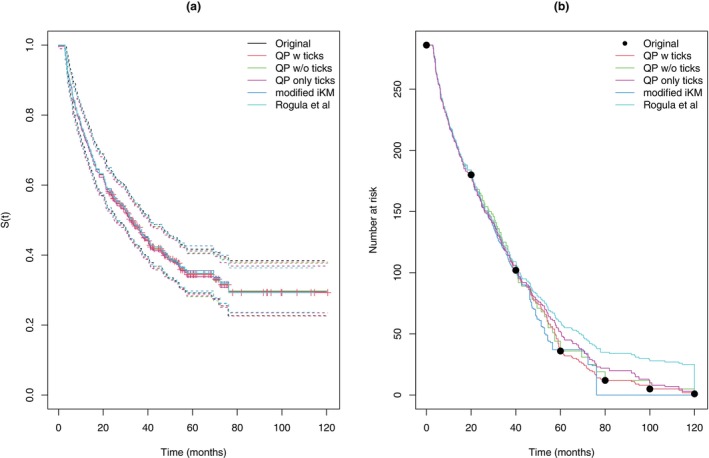
Progression‐free survival estimates (panel a) and estimated number at risk (panel b) from reconstructed pseudo‐IPD using different methods.

The proposed QP method is compared with the corresponding reconstructions using the modified iKM algorithm and the algorithm of Rogula et al. Figure 1 shows the resulting Kaplan–Meier estimates and associated pointwise 95% confidence intervals (in each case these are computed assuming the sampling distribution of $\log\hat{S}\left(t\right)$ is approximately normally). All methods give reasonably good agreement with respect to the implied Kaplan–Meier curve. Moreover, all methods except those not using the persons at risk information give good agreement with the confidence intervals, with deviations occurring later in follow‐up. There is more disagreement regarding the estimated numbers at risk. Notably, the modified iKM algorithm censors individuals at the mid‐point between observed event times, or at the final event time. However, since the last uncensored event occurs at around 76 months, all remaining patients are incorrectly censored at this time, which contradicts the persons at risk information provided. The method of Rogula et al. uses the information from the marked censoring times, but not the information of numbers at risk. As a consequence, the estimated number at risk at later time points is greatly over‐estimated. The corresponding QP estimate using only the marked censoring times also over‐estimates the number at risk at later times, but to a much lesser degree. The total number of events was not reported and there is some disagreement across methods with 177, 178, 181, 172, and 182 estimated for QP with ticks, QP w/o ticks, QP ticks only, modified iKM and Rogula et al., respectively.

Often the purpose of obtaining pseudo‐IPD is to facilitate survival extrapolation for cost‐effectiveness analysis in order to assist the calculation of life expectancy or healthy life expectancy, which usually involves fitting appropriate parametric models. While often an unavoidable part of cost‐effectiveness analysis, in practice survival extrapolation requires careful consideration. For instance, any extrapolation model should be clinically plausible, the sensitivity of conclusions to modeling assumptions should be assessed, and external information should be used in the extrapolation, where possible [23]. Here we consider a simplified process where a Royston–Parmar flexible spline model [24] with one internal knot is fitted to the reconstructed pseudo‐IPD based on each method. This models the log cumulative hazard as a natural cubic spline with respect to log‐time such that

(10) $$\begin{array}{cc} \log\left\{-\log S\left(t\right)\right\} & =\gamma_{0}+\gamma_{1}\log t+\gamma_{2}\left\{{\left(\log t-k_{1}\right)}_{+}^{3}-\frac{k_{2}-k_{1}}{k_{2}-k_{0}}\right.\\ & \;\left.\times \;{\left(\log t-k_{0}\right)}_{+}^{3}-\frac{k_{1}-k_{0}}{k_{2}-k_{0}}{\left(\log t-k_{2}\right)}_{+}^{3}\right\}, \end{array}$$

where $k_{0}$ and $k_{2}$ are the lower and upper boundary knot points and $k_{1}$ is the internal knot point, all measured with respect to log‐time and ${\left(x\right)}_{+}=\max\left(0,x\right)$. When $\gamma_{2}=0$, the parameters $\gamma_{0}$ and $\gamma_{1}$ correspond to the log‐rate and shape in a Weibull model, with $\gamma_{2}\neq$ determining the degree of deviation away from the Weibull trend.

For the QP method without the information on marked censoring times, we note that, although the QP procedure assigns censoring times at either decrement times or the times at which numbers of patients at risk were reported, the published data would not change if the censoring in fact occurred anywhere in the interval $\left(t_{i},t_{i+1}\right]$. As a consequence, when fitting parametric models the censoring times are taken at the midpoint of this interval, that is $0.5\left(t_{i}+t_{i+1}\right)$. This approach matches how the modified iKM algorithm assigns censoring times. All models are fitted using the *flexsurv* package in ***R*** [25]. For comparability of the parameters, the same set of knot points (including boundary knots) are used across all fits. Specifically, we use the default method of choosing knot points in *flexsurv* to choose the knots based on the QP with ticks reconstruction which gives knots at the logged values of t=(0.05,14.67,76.16) corresponding to the minimum, median and maximum observed event time in the pseudo‐IPD based on the QP reconstruction using all the available information.

**FIGURE 2:**
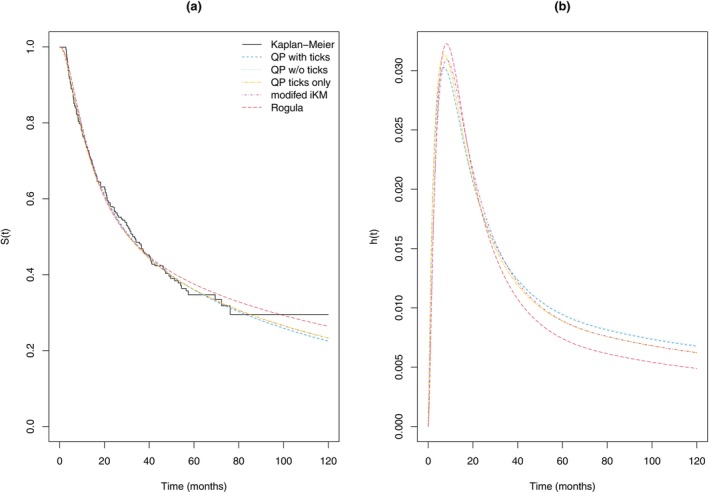
Comparison of Royston–Parmar flexible spline model fitted survivor functions (panel a) and hazard functions (panel b) based on pseudo‐IPD generated via different methods.

Table 1 gives the parameter estimates and their corresponding standard errors for each of the methods. There is close agreement between the estimated parameters for the QP methods. Figure 2 gives the corresponding estimates of survival and the estimated hazard functions. The QP estimates with or without tick marks are virtually indistinguishable. The modified iKM's pseudo‐IPD gives a slightly higher survival estimate (and lower hazard estimate) in the tail which is similar to that given by the QP estimate using just the marked censoring times, while the estimate based on Rogula et al.'s method is noticeably higher.

**TABLE 1:** Comparison of parameter estimates for a Royston–Parmar flexible spline model with one internal knot point.

Parameter	Point estimates			
	QP w ticks	QP w/o ticks	Modified iKM	Rogula
*R* $\gamma_{0}$	−4.8283	−4.8164	−5.3570	−5.4805
$\gamma_{1}$	2.4841	2.4734	2.9415	3.1617
$\gamma_{2}$	0.0693	0.0689	0.0869	0.0976

Parameter	Standard errors			
	QP w ticks	QP w/o ticks	Modified iKM	Rogula
$\gamma_{0}$	0.4032	0.4020	0.4540	0.4569
$\gamma_{1}$	0.3607	0.3589	0.4045	0.3906
$\gamma_{2}$	0.0147	0.0146	0.0164	0.0153

*Note:* The parameters are defined in (10).

For a health economic evaluation it may be necessary to determine the restricted mean survival for these patients up to say 25 years (300 months). Based on extrapolating the Royston–Parmar model fits, while the model based on the pseudo‐IPD from the QP with all data gives an estimate of 6.37 years of progression‐free survival, the modified iKM gives 6.56 years and Rogula et al.'s method gives 7.25 years.

In Section S2 of Supporting Information, the reported progression‐free survival for the two arms of the KEYNOTE‐177 trial is used to provide a comparison of the reconstruction methods with respect to their ability to replicate the reported hazard ratio.

### 4.2 Competing Risks Analysis

Among patients treated with “watch and wait” management, a competing risks analysis was performed with respect to the two component outcomes that defined progression‐free survival; “Treatment or follicular lymphoma related death” and “death from unrelated cause before progression.” The cumulative incidence functions are presented including the number of patients at risk at 20 month intervals and tick marks indicating the timing of censoring. Pointwise 95% confidence intervals for the cumulative incidence of treatment or follicular lymphoma related death are also given.

As before, digitization of the curves was performed by using WebPlotDigitizer [22], identifying 84 and 12 distinct increment points for the two curves, along with 78 tick marks.

**FIGURE 3:**
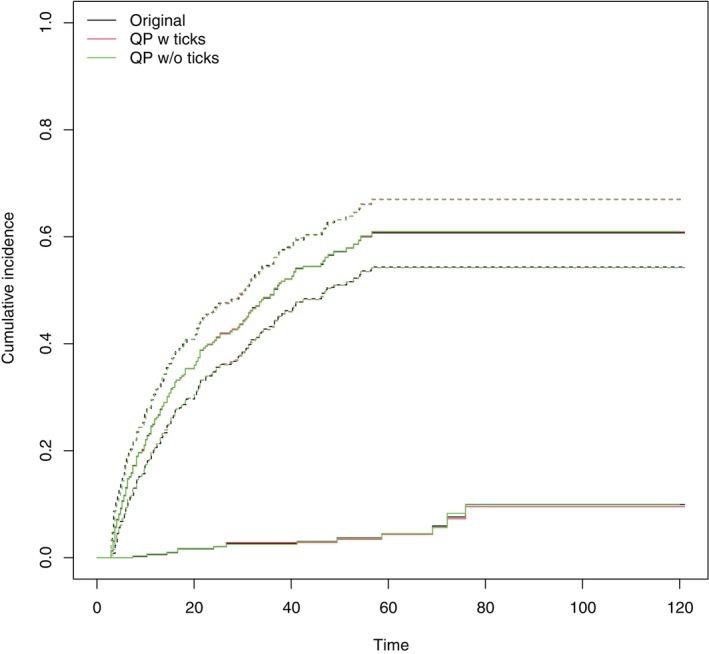
Reconstructed cumulative incidence estimates from the FL study. The upper curve corresponds to treatment or FL‐related death and shows the 95% confidence interval. The lower curve corresponds to death from an unrelated cause before progression.

Figure 3 presents the original digitized and the reconstructed estimates of the cumulative incidence functions. The reconstructions both with and without the information on censoring times give good agreement with the digitized cumulative incidence curve for ‘Treatment or follicular lymphoma related death’. Both reconstructions have some level of disagreement for the cumulative incidence of death from other causes before progression. This is likely to be due to quality of the digitization—the original curve is dashed making identification of step points more difficult.

## 5 Simulations

In this section, three sets of simulations are presented, where the first two sets aim to compare the proposed methods to existing methods with respect to reconstructing IPD for Kaplan–Meier curves, and the last set assesses the proposed methods ability to reconstruct IPD from published cumulative incidence curves.

### 5.1 Comparison With the Modified iKM and Rogula Et Al. Methods

The first set of simulations aim to compare the proposed approach for reconstructing IPD for Kaplan–Meier curves with existing methods. Specifically, the focus is on assessing each method's ability to replicate the results of analyses on the underlying true IPD. A secondary aim is to assess the impact of additional information, such as the marked censoring times, total number of events and reporting of number of patients at risk, on the quality of the reconstruction. A further aim is to assess whether the exact MIQP approach yields more accurate reconstructions than the heuristic QP method based on rounding.

#### 5.1.1 Data Generating Mechanisms

The underlying survival times are generated from a Weibull distribution with hazard function $h\left(t\right)=\lambda^{\alpha }\alpha t^{\alpha -1}$ with a shape parameter α=0.8 and rate parameter λ=0.2. A shape parameter of α=0.8 is chosen to induce a higher rate of events close to time 0 leading to a higher rate of tied events in the coarsened data. The censoring distribution is taken to be independent Unif(2,8), resulting in a censoring rate of around 39%, giving around 76 uncensored deaths per dataset, where a total sample size of 125 patients is assumed. To emulate the level of accuracy that might be observed in practice (for instance reporting to the nearest day or week), the continuous‐time event times are coarsened by rounding up to the next multiple of 0.05. The true IPD is assumed to be reported at this level of resolution. In addition, to emulate the level of error that might occur through digitization, the values of $\hat{S}\left(t\right)$ are rounded to three decimal places when supplied to the algorithms.

Different scenarios are generated by varying the level of auxiliary information available in addition to the Kaplan–Meier curve itself for a given simulated dataset. In the full information case, it is assumed that the number at risk is reported at t=0,1,2,…,8, that the total number of events is given and the set of marked censoring times are available. To provide a direct comparison with the modified iKM algorithm, a scenario where the marked censoring times are omitted is considered. Similarly, to compare with the Rogula et al. method, a scenario is included where only the total sample size and the marked censoring times are used. An intermediate scenario where the marked censoring times, total sample size and total events are known, but not intermediate numbers at risk is also considered for the proposed QP method.

#### 5.1.2 Estimands and Performance Measures

The comparator methods are assessed with respect to their ability to replicate the actual Kaplan–Meier estimate, $\hat{S}\left(t\right)$, and also their ability to replicate the actual cumulative persons at risk, Y(t), across the total follow‐up period. In addition, the maximum likelihood estimates of the parametric Weibull survival model are assessed for each method.

The performance with respect to the replication of $\hat{S}\left(t\right)$ and Y(t) is assessed with respect to the integrated absolute discrepancy compared to the respective quantities in the true IPD, leading to two measures:

$$\Delta_{S}=\int_{0}^{\tau }|\hat{\hat{S}}\left(t\right)-\hat{S}\left(t\right)|\mathrm{dt}$$

and

$$\Delta_{Y}=\int_{0}^{\tau }|\hat{Y}\left(t\right)-Y\left(t\right)|\mathrm{dt},$$

where $\hat{\hat{S}}\left(t\right)$ and $\hat{Y}\left(t\right)$ are the implied Kaplan–Meier estimate and implied number at risk based on the reconstructed pseudo‐IPD. In each case, τ is taken to be the maximum follow‐up time in the true data. A perfect reconstruction would give $\Delta_{S}=0$ and $\Delta_{Y}=0$.

The performance of the estimates of the Weibull model parameters are assessed with respect to their bias and root‐mean squared error, where these are defined in terms of the discrepancy with the corresponding estimate from the true IPD (not the discrepancy from the true population parameter).

#### 5.1.3 Methods

For the full information scenario, and the scenario with all information except the marked censoring times, the exact MIQP method is applied, using IBM ILOG CPLEX, but with a maximum computation time limited to 60 s for the optimized, and absolute and relative convergence tolerances of $1\times 10^{-8}$ and $1\times 10^{-7}$, respectively. In cases where the objective function for the solution of the MIQP optimization at termination was worse than for the approximate method, the approximate solution was used instead.

The heuristic QP method is applied to all scenarios. The underlying continuous quadratic program is solved using the quadprog package in R. The modified iKM algorithm, as implemented in the R package IPDfromKM [8], is applied in the scenario where numbers of patients at risk is assumed known. The method of Rogula et al., as implemented in the package KMtoIPD [26] is applied in the case where only the total sample size and the marked censoring times are used.

#### 5.1.4 Results

Table 2 presents the results from 1000 simulated datasets. As might be expected, the accuracy of the reconstruction increases with the additional information used across the QP methods. There is also a very slight improvement in accuracy for the MIQP method compared to the integer rounding heuristic solution. However, it is probably not sufficient to justify the additional computational complexity. The MIQP and QP methods based on numbers at risk do discernibly better than the corresponding modified iKM reconstructions that use the same information. It is also worth noting that only 4.5% of simulated datasets resulted in a modified iKM reconstruction that matched the total number at risk and total number of events supplied.

**TABLE 2:** Accuracy of different method of reconstructing pseudo‐IPD from simulated Kaplan–Meier curve data in the base case N=125, S(t) points rounded to 3 decimal places.

Measure	With ticks		Without ticks			Without at risk data		
	MIQP	QP	MIQP	QP	mod iKM	QP ticks + nd	QP ticks	Rogula
Δ(S)	0.0018	0.0026	0.0049	0.0092	0.0422	0.0036	0.0204	0.0192
Δ(Y)	1.0568	1.1045	9.3705	10.2064	23.4115	1.9093	17.7187	24.6555
RMSE(log(λ))	0.0022	0.0019	0.0052	0.0070	0.0419	0.0035	0.0178	0.0286
RMSE(log(α))	0.0014	0.0011	0.0033	0.0043	0.0395	0.0021	0.0151	0.0218

*Note:* 20 possible values of t per time unit.

Abbreviation: nd = number of deaths given.

The QP reconstruction using only the information from tick marks gives a comparable match to the Kaplan–Meier curves to the Rogula et al. approach, but somewhat better estimates of numbers at risk and the Weibull model parameters. It also worth noting that just by adding the additional information on the total number of events/deaths to the QP method using only the marked censoring times, brings the quality of the reconstruction almost into line with those also using the intermediate estimates of time at risk.

Additional simulation results are shown in Supporting Information where either the resolution of the reporting times or the number of decimal places the $\hat{S}\left(t\right)$ values are rounded, was varied. As might be expected, the quality of all reconstructions improved as there were fewer tied events in the data and where there was less rounding error. The same pattern of relative performance between methods is seen in all scenarios. In addition, Table S9 of Supporting Information presents a comparison of the QP method and modified iKM when only the total sample size and total number of events is given. In that case, the quality of the reconstruction is generally worse than using the location of marked times only, but is less sensitive to the presence of ties.

### 5.2 Estimation of Treatment Group Differences

A second set of simulations aim to assess the ability of different methods to reconstruct a pair of Kaplan–Meier curves, corresponding to two arms of a clinical trial, in order to assess the ability to estimate a hazard ratio.

#### 5.2.1 Data Generating Mechanisms

Patients in the control group are simulated from the same Weibull distribution as in the single sample case above. The sample size in each arm is taken as N=125. For the treatment group we assume a Weibull distribution with the same shape parameter (ensuring proportionality) but with a lower hazard (corresponding to a log hazard ratio of 0.5). As before, the exact event times are coarsened by rounding up to the next 0.05 and the S(t) values are the true values rounded to three decimal places. The same scenarios in terms of availability of auxiliary information are used as above.

#### 5.2.2 Estimands and Performance Measures

The methods are assessed in terms of the estimated log hazard ratios (HR) obtained by fitting a Cox proportional hazard model and by fitting a fully‐parametric Weibull proportional hazards model to the pooled pseudo‐IPD obtained through each of the reconstruction methods.

To emulate the estimation of hazard ratios from two Kaplan–Meier curves, data was simulated from two arms of a clinical trial. Both a Cox proportional hazards model and a Weibull proportional hazards model was fitted to the pooled pseudo‐IPD obtained through each of the reconstruction methods. The test statistic of the Grambsch–Therneau test of proportionality based on the scaled Schoenfeld residuals [27] was also computed in each case. Finally, the difference in restricted mean survival times [28] was computed, taking τ=5 as the upper time point.

For each of the estimands the performance is assessed by considering the bias and root mean squared error (RMSE), where in each case error is assessed in relation to the corresponding estimate using the true IPD.

The same set of comparator methods are used as in the first set of simulations except that only the heuristic QP method is considered, rather than the MIQP method.

#### 5.2.3 Results

Table 3 gives the resulting bias and RMSE from 1000 simulated datasets. The QP method using the information from tick points performs exceptionally well, with bias and mean squared errors of 0.002 or less for all quantities except the Grambsch–Therneau test statistic. As in the one‐sample case, the QP method without tick points gives lower root MSE for all quantities compared to the modified iKM method using the same information. The most notable discrepancies occur in the Grambsch–Therneau test statistics. Since the data were simulated from data with proportional hazards, the test statistic has an approximate $\chi_{1}^{2}$ distribution using the true IPD. There is close agreement when the QP method is used with tick marks (bias 0.002, RMSE 0.026), substantially more variability for the QP method without tick marks (bias −0.002, RMSE 0.158). There is some tendency for both the QP method using only information from the tick marks and the corresponding Rogula et al. method to give an inflated Grambsch–Therneau test statistic, and both the modified iKM and Rogula et al. methods have relatively high RMSEs in relation to the statistic for the true IPD, indicating a higher risk of discordance between the conclusions regarding assessments of proportional hazard. As in the single sample case, a QP reconstruction using just the marked censoring times and the total number of events (deaths) gives markedly better accuracy than without the total number of events and is also better than the QP reconstruction using total number of events and intermittently observed numbers at risk.

**TABLE 3:** Bias and RMSE in parameter estimates for two group comparisons based on pseudo‐IPD reconstructed via different methods.

	Bias					
	With ticks	Without ticks		Without at risk data		
	QP	QP	mod iKM	QP ticks + nd	QPticks	Rogula
Cox log(HR)	0.000	−0.002	0.004	0.000	0.001	0.000
Weibull log(HR)	0.000	−0.001	0.001	0.001	−0.002	−0.001
Grambsch–Therneau $X^{2}$	0.002	−0.002	0.058	0.005	0.107	0.164
ΔRMST	0.000	0.001	0.002	0.000	0.001	0.000

	RMSE					
	With ticks	Without ticks		Without at risk data		
	QP	QP	mod iKM	QP ticks + nd	QP ticks	Rogula
Cox log(HR)	0.001	0.006	0.033	0.002	0.014	0.019
Weibull log(HR)	0.001	0.004	0.023	0.002	0.015	0.020
Grambsch–Therneau $X^{2}$	0.026	0.158	0.821	0.045	0.371	0.564
ΔRMST	0.002	0.004	0.008	0.002	0.003	0.004

Abbreviation: nd = total number of deaths given.

### 5.3 Performance of Competing Risks Reconstructions

The final set of simulations aim to confirm the accuracy of the proposed method for reconstructing IPD from cumulative incidence estimates from competing risks data. As above, the focus is on estimating treatment effects, but here the treatment effect could be with respect to either of two competing events and can be either based on the cause‐specific hazard or based on the sub‐distribution hazard using a Fine‐Gray model.

#### 5.3.1 Data Generating Mechanisms

Competing risks data are simulated from a process with two competing risks and two treatment groups.

Let Z=0,1 represent the treatment group, then the competing risks data are generated by assuming the CIF of the first risk, satisfies

$$F_{1}\left(t;Z=0\right)=\kappa \left[1-\exp\left\{-{\left(\lambda_{01}t\right)}^{\alpha_{1}}\right\}\right]$$

and that $F_{1}$ adheres to the Fine‐Gray assumption of proportional sub‐distribution hazards with a log‐hazard ratio of $\beta_{1}$ such that

$$\begin{array}{cc} F_{1}\left(t;Z=1\right) & =1-{\left(1-F_{1}\left(t;Z=0\right)\right)}^{\exp\beta_{1}}\\ & =1-{\left[1-\kappa \left\{1-\exp\left(-{\left(\lambda_{01}t\right)}^{\alpha_{1}}\right)\right\}\right]}^{\exp\beta_{1}}. \end{array}$$

To allow $F_{2}\left(t;Z\right)$ to be a valid CIF, we note that the model implies $\lim_{t\to 0}F_{1}\left(t;Z\right)=1-{\left(1-\kappa \right)}^{\exp\left(\beta_{1}Z\right)}$, so then take 


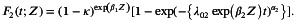




Hence, the Fine‐Gray model for cause 1 is correctly specified, while for the Fine‐Gray model for cause 2 and for either of the Cox models with respect to the cause‐specific hazards, the models are miss‐specified.

Specifically, we take $\left(\kappa ,\lambda_{01},\alpha_{1},\lambda_{02},\alpha_{2},\beta_{1},\beta_{2}\right)=\left(0.6,0.4,1.2,0.2,1.5,-0.3,0.3\right).$ The censoring distribution is assumed to be Unif(1,6). The resolution of reporting of times is again taken as the nearest 0.05 and each treatment group has N=125 patients. For each simulated dataset, the Aalen‐Johansen estimates of the cumulative incidence functions are computed for both of the competing events, where the CIF points are rounded to three decimal places to emulate digitization error.

Three scenarios are generated by varying the amount of auxiliary information supplied to the algorithm: use of marked censoring times, numbers at risk at times 1,2,…,5 and total number of events of each type; use of only numbers at risk at times 1,2,…,5 and total number of events of each type; use of only the marked censoring times.

#### 5.3.2 Estimands and Performance Measures

For each of the scenarios, the Fine‐Gray sub‐distribution hazard ratio, and the cause‐specific hazard ratio is estimated for both of the competing events. In each case, performance is assessed based on bias and RMSE defined with respect to the corresponding estimates obtained using the true simulated IPD.

#### 5.3.3 Results

Table 4 displays the results of 1000 simulated datasets, where for each scenario the heuristic QP method is used. In all cases, the reconstructed data produces estimates close to those found using the original data. As one would expect, there is a larger discrepancy between the estimates from the true IPD when less information is used in the reconstruction.

**TABLE 4:** Bias and RMSE in parameter estimates for competing risks analyses.

	Bias		
	QP with ticks	QP w/o ticks	QP only ticks
Fine‐Gray HR_1_	−0.001	0.001	−0.001
Fine‐Gray HR_2_	−0.001	0.000	−0.010
CSH HR_1_	−0.001	0.000	0.006
CSH HR_2_	−0.001	−0.001	−0.008

	RMSE		
	QP with ticks	QP w/o ticks	QP only ticks
Fine‐Gray HR_1_	0.001	0.003	0.026
Fine‐Gray HR_2_	0.001	0.003	0.037
CSH HR_1_	0.002	0.008	0.025
CSH HR_2_	0.002	0.011	0.031

## 6 Discussion

The results in the paper have confirmed previous work [6, 9] that has shown that accurate pseudo‐IPD can be reconstructed from published Kaplan–Meier curves, provided either inteim information of numbers of patients at risk or the marked censoring times are available. It also shows that some gain in accuracy can be achieved by adopting a quadratic programming approach, which treats the numbers of patients at risk as strict equality constraints. Moreover, the quadratic programming approach easily accommodates both the information on individual censoring times and the numbers at risk, which was shown to give a more appreciable improvement in accuracy, particularly if the pseudo‐IPD is to be used for a parametric analysis.

A limitation of the simulation study in Section 5.1 is that the underlying model for data generation is restricted to a particular Weibull distribution. The non‐parametric nature of the Kaplan–Meier method should mean that the patterns seen in the performance measures $\Delta_{S}$ and $\Delta_{R}$ would likely translate to other underlying distributions. However, it is possible other models might behave differently in terms of the discrepancy between the IPD and reconstructed estimates of the model parameters.

The proposed quadratic programming approach also allows for reconstruction of competing risks survival data from published cumulative incidence curves. This could be particularly useful in performing meta‐analyses in cases where, for instance, some studies report the Fine‐Gray sub‐distribution hazard ratio and other studies report the hazard ratio associated with the cause‐specific hazard, or report neither. Existing methods for meta‐analysis for competing risks events rely on strong assumptions such as constant hazards [29], or only use information on the event counts and total follow‐up in the studies [30].

An ***R*** package, *CIFresolve*, has been prepared to implement the QP‐based methods proposed in the paper. For the integer rounding approach, the functions use the *quadprog* package to solve the continuous quadratic program. The MIQP approach can also be used via the *Rcplex* package which allows IBM CPLEX to be called from ***R***, provided it is installed. The package is available via the author's Github page; https://github.com/andrewtitman/CIFresolve.

While the optimization problem in the proposed method is strictly a MIQP, simulation results indicate the additional computational burden of using methods for MIQP compared to simple integer rounding of the continuous solution is usually not justified. Hence using the quicker and widely available approach based on standard QP is recommended in most cases. If the total patient time at risk, $t_{P}$ has been given (or can be inferred, for instance via the MLE of an exponential model) then this implies an equality constraint; $t_{P}=\sum_{i}t_{i}\left(d_{i}+c_{i}\right)$. In practice, since the exact censoring times may not be known and the decrement points may have been digitized, the information would be better incorporated by stipulating;

$$\left(1-\delta_{P}\right)t_{P}\leq \sum_{i}t_{i}\left(d_{i}+c_{i}\right)\leq \left(1+\delta_{P}\right)t_{P}$$

for a suitably chosen $\delta_{P}$, for instance, $\delta_{P}=0.001$. However, it is not clear how a heuristic rounding rule (such as in (5)) could be stipulated to preserve these inequalities when going from a continuous to integer solution of the QP. Hence, the MIQP approach to optimization is needed to incorporate these constraints.

The methods can also be adapted to provide pseudo‐IPD from published Nelson‐Aalen estimates of the cumulative hazard, or cumulative cause‐specific hazard. In that case, the *increment* of the Nelson‐Aalen estimate at time $t_{i}$ can be used as $o_{i}$ or $o_{\mathrm{ij}}$ in (3) and (9), with the methods otherwise proceeding the same. Similarly, the methods can be adapted to account for other sources of additional information. For instance, some Kaplan–Meier or cumulative incidence function plots include intermittently‐reported cumulative event counts, in addition to numbers at risk.

A further extension is to allow reconstruction of non‐parametric survival curves estimated on left‐truncated and right‐censored data using the Lynden‐Bell generalization of the Kaplan–Meier estimator [31], provided information on the, not necessarily decreasing, numbers at risk is available intermittently. In that case, it is not possible to infer individual‐level data, but the sufficient statistics for parametric models under the standard assumption that the left‐truncation times are quasi‐independent of survival (aggregated numbers at risk and numbers of events over time) should be recoverable.

Recently in oncology health technology assessments, there is interest in fitting multi‐state illness‐death models using information from published progression‐free survival and overall survival curves [32, 33]. An important step in this process is ensuring good reconstructions of the pseudo‐IPD from the PFS and OS curves. Potentially, the proposed QP approach could be used to ensure consistency between the sources of information. In particular, assuming the PFS and OS curves come from the same set of patients and that each patient's right‐censoring time is the same for PFS and OS, the number of patients at risk in the PFS data must necessarily be less than equal to that for OS at all follow‐up times. Similarly, the number of patients censored for OS must be greater than or equal to the number of PFS censorings at all times. These constraints can be incorporated into relevant linear constraints in the QP provided care is taken to align the decrement points and marked censoring times on the digitized PFS and OS curves.

In some cases, the published Kaplan–Meier curve may provide no person‐at risk information or marked censoring times, but will include pointwise 95% confidence intervals. If the method by which the confidence intervals have been constructed is known or can be inferred, it may be possible to gain additional information on the number of patients at risk, which could be incorporated into the objective function. For instance, if Greenwood's formula has been used for the standard error and naive (symmetric) asymptotic 95% confidence intervals have been constructed, then the limits will be of the form

$$\hat{S}\left(t\right)\left\{1\pm 1.96\sqrt{\sum_{i:t_{i}\leq t}\frac{d_{i}}{r_{i}\left(r_{i}-d_{i}\right)}}\right\}.$$

In theory, digitization could be used to infer $\frac{d_{i}}{r_{i}\left(r_{i}-d_{i}\right)}$ from the change in confidence intervals at decrements of $\hat{S}\left(t\right)$. However, the image quality is unlikely to be sufficient to capture this accurately since it is of order $n^{-2}$, at a given decrement of $\hat{S}\left(t\right)$. Nevertheless, it may be possible to incorporate information on the implied values of $\sum_{i:t_{i}\leq t}\frac{d_{i}}{r_{i}\left(r_{i}-d_{i}\right)}$ at suitably spaced time points. Optimization in this case is more challenging since either the objective function or one or more of the constraints would need to be non‐linear.

## Funding

The author has nothing to report.

## Conflicts of Interest

The author declares no conflicts of interest.

## Supporting information


**Data S1:** sim70474‐sup‐0001‐Supinfo.pdf.

## Data Availability

Data sharing is not applicable to this article as no new data were created or analyzed in this study.

## Appendix A Form of the Quadratic Program

Let H be an $N_{s}\times 2N_{s}$ matrix where

$${\left\{H\right\}}_{\mathrm{ij}}=\left(\begin{array}{ll} o_{i} & \text{if}\;j<i\;\text{or}\;N_{s}<j<i+N_{s}\\ 1 & \text{if}\;j=i\\ 0 & \text{otherwise}. \end{array}\right.$$

Then, minimizing D in (3) with respect to $x={\left(d_{1},d_{2},\ldots ,d_{N_{s}},c_{1},c_{2},\ldots ,c_{N_{s}}\right)}^{\prime }$ is equivalent to minimizing $\frac{1}{2}x^{\prime }\mathrm{Qx}+q^{\prime }x$ where $Q=H^{\prime }H$ and $q=-o_{N}^{\prime }H$ where $o_{N}$ is a vector of length $N_{s}$ with *i*th entry $N\times o_{i}$.

The constraint matrix A and vector b depend on the specific auxiliary information provided, but A is an $N_{c}\times 2N_{s}$ matrix and the *j*th row of A defines either an equality or inequality constraint bounded by the corresponding entry of b, which is a vector of length $N_{c}$.

A similar formulation can be constructed for D given in (9).

## References

[1] R. D. Riley , P. C. Lambert , and G. Abo‐Zaid , “Meta‐Analysis of Individual Participant Data: Rationale, Conduct and Reporting,” BMJ 340 (2010): c221. 20139215 10.1136/bmj.c221

[2] M. K. B. Parmar , V. Torri , and L. Stewart , “Extracting Summary Statistics to Perform Meta‐Analyses of the Published Literautre for Survival Endpoints,” Statistics in Medicine 17, no. 24 (1998): 2815–2834. 9921604 10.1002/(sici)1097-0258(19981230)17:24<2815::aid-sim110>3.0.co;2-8

[3] J. F. Tierney , L. A. Stewart , D. Ghersi , S. Burdett , and M. R. Sydes , “Practical Methods for Incorporating Summary Time‐To‐Event Data Into Meta‐Analysis,” Trials 8 (2007): 1–16. 17555582 10.1186/1745-6215-8-16 PMC1920534

[4] Z. Liu , B. Rich , and J. A. Hanley , “Recovering the Raw Data Behind a Non‐Parametric Survival Curve,” Systematic Reviews 3 (2014): 151. 25551437 10.1186/2046-4053-3-151 PMC4293001

[5] M. W. Hoyle and W. Henley , “Improved Curve Fits to Summary Survival Data: Application to Economic Evaluation of Health Technologies,” BMC Medical Research Methodology 11 (2011): 139. 21985358 10.1186/1471-2288-11-139 PMC3198983

[6] P. Guyot , A. E. Ades , M. J. Ouwens , and N. J. Welton , “Enhanced Secondary Analysis of Survival Data: Reconstructing the Data From Published Kaplan–Meier Survival Curves,” BMC Medical Research Methodology 12 (2012): 1. 22297116 10.1186/1471-2288-12-9 PMC3313891

[7] N. Liu , Y. Zhou , and J. J. Lee , “IPDfromKM: Reconstruct Individual Patient Data From Published Kaplan–Meier Survival Curves,” BMC Medical Research Methodology 21 (2021): 111. 34074267 10.1186/s12874-021-01308-8 PMC8168323

[8] N. Liu and J. J. Lee , “IPDfromKM: Map Digitized Survival Curves Back to Individual Patient Data. R Package Version 0.1.10,” 2020.

[9] B. Rogula , G. Lozano‐Oretga , and K. M. Johnston , “A Method for Reconstructing Individual Patient Data From Kaplan–Meier Survival Curves That Incorporate Marked Censoring Times,” MDM Policy and Practice 7, no. 1 (2022): 1–8. 10.1177/23814683221077643 PMC8808036 35128059

[10] H. Putter , M. Fiocco , and R. B. Geskus , “Tutorial in Biostatistics: Competing Risks and Multi‐State Models,” Statistics in Medicine 26, no. 11 (2007): 2389–2430. 17031868 10.1002/sim.2712

[11] D. Goldfarb and A. Idnani , “A Numerically Stable Dual Method for Solving Strictly Convex Quadratic Programs,” Mathematical Programming 27, no. 1 (1983): 1–33.

[12] A. Berwin , R. Turlach , and A. Weingessel , “*quadprog*: Functions to Solve Quadratic Programming Problems. R Package Version 1.5‐7,” 2019.

[13] M. A. Duran and I. E. Grossmann , “An Outer‐Approximation Algorithm for a Class of Mixed‐Integer Nonlinear Programs,” Mathematical Programming 36 (1986): 307–339.

[14] O. K. Gupta and A. Ravindran , “Branch and Bound Experiments in Convex Nonlinear Integer Programming,” Management Science 31, no. 12 (1985): 1533–1546.

[15] IBM ILOG CPLEX , Version 12.1: Users Manual for CPLEX (International Business Machines Corporation, 2009).

[16] H. Corrada Bravo and S. Theussl , “Rcplex: R Interface to CPLEX. R Package Version 0.3‐3,” 2016.

[17] M. Roettger , G. Gelius‐Dietrich , and C. J. Fritzemeier , “cplexAPI: R Interface to C API of IBM ILOG CPLEX. R Package Version 1.3.6,” 2019.

[18] R. Kannan and C. L. Monma , “On the Computational Complexity of Integer Programming Problems,” in Optimization and Operations Research: Proceedings of a Workshop Held at the University of Bonn, October 2–8, 1977 (Springer Berlin Heidelberg, 1978), 161–172.

[19] R. L. Prentice , J. D. Kalbfleisch , J. A. V. Peterson , N. Flournoy , V. T. Farewell , and N. E. Breslow , “The Analysis of Failure Times in the Presence of Competing Risks,” Biometrics 34 (1978): 541–554. 373811

[20] J. P. Fine and R. J. Gray , “A Proportional Hazards Model for the Subdistribution of a Competing Risk,” Journal of the American Statistical Association 94 (1999): 496–509. 10.1080/01621459.2017.1401540 PMC6502476 31073256

[21] T. C. El‐Galaly , A. E. Bilgrau , P. de Nully Brown , et al., “A Population‐Based Study of Prognosis in Advanced Stage Follicular Lymphoma Managed by Watch and Wait,” British Journal of Haematology 169, no. 3 (2015): 435–444. 25709094 10.1111/bjh.13316

[22] A. Rohatgi , “WebPlotDigitizer, Version 5,” (2022), http://automeris.io/wpd/.

[23] N. Latimer , “NICE DSU Technical Support Document 14: Undertaking Survival Analysis for Economic Evaluations Alongside Clinical Trials – Extrapolation With Patient‐Level Data,” (2011), http://www.nicedsu.org.uk. 27905716

[24] P. Royston and M. K. B. Parmar , “Flexible Parametric Proportional‐Hazards and Proportional‐Odds Models for Censored Survival Data, With Application to Prognostic Modelling and Estimation of Treatment Effects,” Statistics in Medicine 21, no. 15 (2002): 2175–2197. 12210632 10.1002/sim.1203

[25] C. H. Jackson , “Flexsurv: A Platform for Parametric Survival Modeling in R,” Journal of Statistical Software 70, no. 8 (2016): 1–33. 10.18637/jss.v070.i08 PMC5868723 29593450

[26] B. Rogula , “KMtoIPD: Reconstruct Individual Patient Data From Kaplan–Meier Survival Curves. R Package Version 1.0.0,” 2025.

[27] P. M. Grambsch and T. M. Therneau , “Proportional Hazards Tests and Diagnostics Based on Weighted Residuals,” Biometrika 81, no. 3 (1994): 515–526.

[28] P. Royston and M. K. B. Parmar , “Restricted Mean Survival Time: An Alternative to the Hazard Ratio for the Design and Analysis of Randomized Trials With a Time‐To‐Event Outcome,” BMC Medical Research Methodology 13 (2013): 152. 24314264 10.1186/1471-2288-13-152 PMC3922847

[29] F. Bonofiglio , J. Beyersmann , M. Schumacher , M. Koller , and G. Schwarzer , “Meta‐Analysis for Aggregated Survival Data With Competing Risks: A Parametric Approach Using Cumulative Incidence Functions,” Research Synthesis Methods 7, no. 3 (2016): 282–293. 26387882 10.1002/jrsm.1165

[30] A. E. Ades , I. Mavranezouli , S. Dias , N. J. Welton , C. Whittington , and T. Kendall , “Network Meta‐Analysis With Competing Risk Outcomes,” Value in Health 13, no. 8 (2010): 976–983. 20825617 10.1111/j.1524-4733.2010.00784.x

[31] D. Lynden‐Bell , “A Method of Allowing for Known Observational Selection in Small Samples Applied to 3CR Quasars,” Monthly Notices of the Royal Astronomical Society 155, no. 1 (1971): 95–118.

[32] J. P. Jansen , D. Incerti , and T. A. Trikalinos , “Multi‐State Network Meta‐Analysis of Progression and Survival Data,” Statistics in Medicine 42, no. 19 (2023): 3371–3391. 37300446 10.1002/sim.9810 PMC10865415

[33] M. A. Pahuta , J. Werier , E. K. Wai , R. A. Patchell , and D. Coyle , “A Technique for Approximating Transition Rates From Published Survival Analyses,” Cost Effectiveness and Resource Allocation 17 (2019): 12. 31303865 10.1186/s12962-019-0182-7 PMC6604134

