# Supplementary material for: Using Quadratic Programming to Reconstruct Data From Published Survival and Competing Risks Analyses
Source: Stat Med. 2026 Mar 3;45(6-7):e70474. doi: 10.1002/sim.70474 (PMC12956426; doi:10.1002/sim.70474)
Supplement: Supplementary file 1 — Data S1: sim70474‐sup‐0001‐Supinfo.pdf. [file SIM-45-0-s001.pdf]

Supplementary Material: Using quadratic programming to reconstruct data from published survival and competing risks analyses

Andrew C. Titman*

School of Mathematical Sciences

Lancaster University, UK

S1 Additional Simulation Results

This section provides results for alternative scenarios similar to those presented in Section 5.1 of the main paper. Specifically, the same Weibull distribution is used to generate the survival times and the same censoring distribution is used. However, the resolution at which event times are reported is varied between 5 times per time unit (i.e. rounded up in increments of 0.2) to 80 times per time unit (rounded up in increments of 0.0125). Similarly $\hat{S}(t)$ is rounded to either 3 or 4 decimal places. These complement the base case considered in the main paper where $\hat{S}(t)$ is rounded to 3 decimal places and times are reported in increments of 0.05. In all these scenarios, the number of patients is fixed at $N = 125$. However, as a more extreme scenario we consider $N = 500$ for the most extreme case (increments of 0.2 and rounded to 3 decimal places). Note that the relative effect of rounding $\hat{S}(t)$ increases with the sample size since there is a greater range of possible decrement values. The results are given in Tables S1-S8. In Table S9, scenarios where only the total sample size and total number of events are presented, with the number of reporting times per time unit varied and the accuracy of the QP method compared to the modified iKM algorithm.

*email: a.titman@lancaster.ac.uk

Table S1: Accuracy of different method of reconstructing pseudo-IPD from simulated Kaplan-Meier curve data in the base case ($N = 125$, $S(t)$ points rounded to 4 decimal places. 80 possible values of t per time unit). nd = number of deaths given.

	With ticks		Without ticks			Without at risk data		
Measure	MIQP	QP	MIQP	QP	mod iKM	QP ticks + nd	QP ticks	Rogula
$\Delta(S)$	0.0000	0.0002	0.0047	0.0067	0.0269	0.0004	0.0207	0.0208
$\Delta(Y)$	0.2014	0.1975	8.7787	9.2743	19.3608	0.3281	5.9701	7.5593
RMSE($\log(\lambda)$)	0.0009	0.0007	0.0046	0.0050	0.0363	0.0013	0.0100	0.0145
RMSE($\log(\alpha)$)	0.0006	0.0004	0.0028	0.0031	0.0295	0.0008	0.0076	0.0101

Table S2: Accuracy of different method of reconstructing pseudo-IPD from simulated Kaplan-Meier curve data in the base case ($N = 125$, $S(t)$ points rounded to 4 decimal places. 40 possible values of t per time unit). nd = number of deaths given.

	With ticks		Without ticks			Without at risk data		
Measure	MIQP	QP	MIQP	QP	mod iKM	QP ticks + nd	QP ticks	Rogula
$\Delta(S)$	0.0001	0.0006	0.0044	0.0076	0.0342	0.0010	0.0208	0.0213
$\Delta(Y)$	0.3675	0.4017	8.7250	9.3761	21.4586	0.6529	10.3333	13.3759
RMSE($\log(\lambda)$)	0.0013	0.0010	0.0044	0.0052	0.0415	0.0018	0.0131	0.0192
RMSE($\log(\alpha)$)	0.0009	0.0006	0.0028	0.0034	0.0339	0.0012	0.0105	0.0143

Table S3: Accuracy of different method of reconstructing pseudo-IPD from simulated Kaplan-Meier curve data in the base case ($N = 125$, $S(t)$ points rounded to 4 decimal places. 20 possible values of t per time unit). nd = number of deaths given.

	With ticks		Without ticks			Without at risk data		
Measure	MIQP	QP	MIQP	QP	mod iKM	QP ticks + nd	QP ticks	Rogula
$\Delta(S)$	0.0004	0.0013	0.0044	0.0089	0.0413	0.0019	0.0199	0.0192
$\Delta(Y)$	0.7026	0.8476	9.0059	9.9058	23.0108	1.3555	17.3816	24.0909
RMSE($\log(\lambda)$)	0.0018	0.0016	0.0055	0.0067	0.0432	0.0031	0.0163	0.0280
RMSE($\log(\alpha)$)	0.0012	0.0010	0.0035	0.0043	0.0414	0.0020	0.0135	0.0212

Table S4: Accuracy of different method of reconstructing pseudo-IPD from simulated Kaplan-Meier curve data in the base case ($N = 125$, $S(t)$ points rounded to 3 decimal places. 80 possible values of t per time unit). nd = number of deaths given.

	With ticks		Without ticks			Without at risk data		
Measure	MIQP	QP	MIQP	QP	mod iKM	QP ticks + nd	QP ticks	Rogula
$\Delta(S)$	0.0009	0.0011	0.0052	0.0063	0.0270	0.0018	0.0213	0.0206
$\Delta(Y)$	0.3805	0.3407	8.8835	9.3471	19.2178	0.6502	5.5274	7.6200
RMSE($\log(\lambda)$)	0.0014	0.0011	0.0046	0.0050	0.0343	0.0020	0.0110	0.0145
RMSE($\log(\alpha)$)	0.0008	0.0006	0.0028	0.0030	0.0281	0.0011	0.0080	0.0100

Table S5: Accuracy of different method of reconstructing pseudo-IPD from simulated Kaplan-Meier curve data in the base case ($N = 125$, $S(t)$ points rounded to 3 decimal places. 40 possible values of t per time unit). nd = number of deaths given.

	With ticks		Without ticks			Without at risk data		
Measure	MIQP	QP	MIQP	QP	mod iKM	QP ticks + nd	QP ticks	Rogula
$\Delta(S)$	0.0014	0.0018	0.0050	0.0074	0.0343	0.0028	0.0214	0.0215
$\Delta(Y)$	0.6536	0.6289	8.9792	9.6367	21.2318	1.1509	10.1985	13.4374
RMSE($\log(\lambda)$)	0.0017	0.0015	0.0048	0.0061	0.0398	0.0028	0.0159	0.0209
RMSE($\log(\alpha)$)	0.0010	0.0008	0.0030	0.0037	0.0320	0.0016	0.0124	0.0153

Table S6: Accuracy of different method of reconstructing pseudo-IPD from simulated Kaplan-Meier curve data in the base case ($N = 125$, $S(t)$ points rounded to 3 decimal places. 10 possible values of t per time unit). nd = number of deaths given.

	With ticks		Without ticks			Without at risk data		
Measure	MIQP	QP	MIQP	QP	mod iKM	QP ticks + nd	QP ticks	Rogula
$\Delta(S)$	0.0023	0.0035	0.0046	0.0102	0.0495	0.0047	0.0189	0.0164
$\Delta(Y)$	1.5415	1.7821	9.4089	10.4875	23.6824	2.9974	25.7011	41.2155
RMSE($\log(\lambda)$)	0.0027	0.0024	0.0080	0.0092	0.0396	0.0043	0.0208	0.0436
RMSE($\log(\alpha)$)	0.0018	0.0015	0.0050	0.0059	0.0470	0.0029	0.0191	0.0337

Table S7: Accuracy of different method of reconstructing pseudo-IPD from simulated Kaplan-Meier curve data in the base case ($N = 125$, $S(t)$ points rounded to 3 decimal places. 5 possible values of t per time unit). nd = number of deaths given.

	With ticks		Without ticks			Without at risk data		
Measure	MIQP	QP	MIQP	QP	mod iKM	QP ticks + nd	QP ticks	Rogula
$\Delta(S)$	0.0024	0.0048	0.0040	0.0111	0.0573	0.0077	0.0175	0.0144
$\Delta(Y)$	1.8750	2.5502	9.4424	11.0680	24.8116	4.5896	33.9814	64.9436
RMSE($\log(\lambda)$)	0.0032	0.0031	0.0148	0.0151	0.0361	0.0060	0.0284	0.0651
RMSE($\log(\alpha)$)	0.0023	0.0022	0.0097	0.0103	0.0647	0.0045	0.0280	0.0526

Table S8: Accuracy of different method of reconstructing pseudo-IPD from simulated Kaplan-Meier curve data in the base case ($N = 500$, $S(t)$ points rounded to 3 decimal places. 10 possible values of t per time unit). nd = number of deaths given.

	With ticks		Without ticks			Without at risk data		
Measure	MIQP	QP	MIQP	QP	mod iKM	QP ticks + nd	QP ticks	Rogula
$\Delta(S)$	0.0025	0.0040	0.0037	0.0069	0.0270	0.0050	0.0055	0.0033
$\Delta(Y)$	6.6974	8.5122	16.4190	20.2670	46.4180	14.3748	164.2274	424.0880
RMSE($\log(\lambda)$)	0.0019	0.0018	0.0145	0.0144	0.0102	0.0026	0.0222	0.0713
RMSE($\log(\alpha)$)	0.0013	0.0013	0.0093	0.0090	0.0622	0.0019	0.0176	0.0546

Table S9: Accuracy of QP method compared to modified iKM when only total sample size and total number of events is given ($N = 125$, $S(t)$ points rounded to 3 decimal places. Varying the resolution of the time reporting.).

Points per unit	40		20		10		5	
Measure	QP	mod iKM	QP	mod iKM	QP	mod iKM	QP	mod iKM
$\Delta(S)$	0.0225	0.0457	0.0232	0.0529	0.0218	0.0621	0.0210	0.0690
$\Delta(Y)$	14.5357	40.8131	16.9267	43.3470	19.8306	44.0137	24.2940	45.4036
RMSE($\log(\lambda)$)	0.0110	0.0371	0.0107	0.0412	0.0112	0.0441	0.0168	0.0428
RMSE($\log(\alpha)$)	0.0058	0.0278	0.0071	0.0359	0.0085	0.0484	0.0133	0.0700

S2 Additional Example: PFS in the KEYNOTE-177 trial

To provide an example that allows for a real-world assessment of the methods with respect to a reported Cox proportional hazards model hazard ratio, the analysis of progression-free survival for pembrolizumab compared to chemotherapy in the KEYNOTE-177 trial is used (Figure 1 in Andre et al.[1]). There is relatively detailed reporting, with numbers of risk reported every four months over 44 months of follow-up, and marked censoring times provided on the plot. The published paper only gives the total number of PFS events across both arms. However, additional information including the number of events in each arm is given in Table 25 of NICE TA709,[2] where the total person-months at risk in each arm is also given. While the person-months is not directly used in the reconstructions, it can be used as an additional way of measuring accuracy.

Table S10 gives the summary measures from the original IPD and using pseudo-IPD from each reconstruction method. The QP-based reconstructions using information on patients at risk and with or without the information from marked censoring times, both give the same HR estimate as the original report up to two decimal places. Constraining the total number of events to be equal to the number reported has little effect on the HR estimate, but further improves the accuracy of the estimate of total time at risk. The modified iKM method also provides a HR estimate close to the original, although there is some under-estimation of total time at risk for the pembrolizumab arm.

The QP method using only the information from marked censoring times performs somewhat worse, over-estimating both events and time at risk for the pembrolizumab arm, although the HR estimate is still fairly close to the one reported. In this case, adding information on the total number of events does not have much effect on the HR estimate, but did lead to the estimates of total time at risk being much closer to those originally reported. Rogula *et al*'s method performed similar to the QP method without ticks but further over-states the total person-time at risk in each arm.

References

1. Andre T, Shiu KK, Kim TW, Jensen BV, et al. Pembrolizumab in microsatellite-instability–high advanced colorectal cancer. *N Engl J Med.* 2020; 383(23):2207-2218.

2. National Institute for Health and Care Excellence. TA709: Pembrolizumab for untreated metastatic colorectal cancer with high microsatellite instability or mismatch repair deficiency. Company evidence submission. 2021.

Table S10: Trial summary measures for KEYNOTE-177 trial based on the original and reconstructed IPD using different methods. te = Total events. * = Events constrained at these numbers by the quadratic program.

Method	HR (95% CI)	Events (Pem)	Events (Chem)	Time at risk (Pem)	Time at risk (Chem)
Original data	0.60 (0.45, 0.80)	82	113	2238.8	1487.3
QP with ticks + te	0.601 (0.450, 0.803)	82*	113*	2240.5	1495.3
QP with ticks	0.599 (0.449, 0.800)	82	116	2240.6	1512.8
QP w/o ticks + te	0.597 (0.447, 0.798)	82*	113*	2222.8	1491.1
QP w/o ticks	0.597 (0.456, 0.807)	84	118	2242.9	1524.0
modified iKM	0.604 (0.451, 0.810)	82	112	2139.2	1472.4
QP only ticks + te	0.590 (0.442, 0.789)	82*	113*	2240.7	1495.5
QP only ticks	0.594 (0.448, 0.788)	85	122	2325.3	1607.1
Rogula et al	0.585 (0.441, 0.777)	85	122	2540.3	1634.4
